# Simultaneous Detection of Influenza A/B, Respiratory Syncytial Virus, and SARS-CoV-2 in Nasopharyngeal Swabs by One-Tube Multiplex Reverse Transcription Polymerase Chain Reaction

**DOI:** 10.3390/tropicalmed8060326

**Published:** 2023-06-19

**Authors:** Bader S. Alotaibi, Bilal Ahmad Tantry, Altaf Bandy, Reyaz Ahmad, Syed Quibtiya Khursheed, Arshid Ahmad, Mohammed Ageeli Hakami, Naveed Nazir Shah

**Affiliations:** 1 Department of Clinical Laboratory Science, College of Applied Medical Sciences, Shaqra University, Alquwayiyah 19257, Saudi Arabia; balotaibi@su.edu.sa (B.S.A.); m.hakami@su.edu.sa (M.A.H.); 2 Department of Microbiology, Government Medical College, Srinagar 190010, India; bilaltantry@gmail.com (B.A.T.); reyaz973khan@gmail.com (R.A.); 3 Department of Community Medicine, College of Medicine, Shaqra University, Shaqra 15273, Saudi Arabia; ahbanday@su.edu.sa; 4 Department of Surgery, SKIMS Medical College, Srinagar 190018, India; drquibtiya@yahoo.co.in; 5 Department of Pulmonary Medicine, Government Medical College, Srinagar 190001, India; arshidehmed2015@gmail.com

**Keywords:** real-time reverse transcription, influenza A, influenza B, RSV, SARS-CoV-2, 5-PLEX assay

## Abstract

The treatment and outcome of respiratory virus infections differ. SARS-CoV-2, as well as other respiratory viruses such as influenza virus (A and B) and respiratory syncytial virus (RSV), require simultaneous, cost-effective, and rapid differential detection. We used a gold standard five-target single-step RT-PCR to detect influenza viruses, RSV, and SARS-CoV-2, and this method can be extended to detect influenza virus subtypes. As a result, this five-target single-step RT-PCR method is ideal for differentiating respiratory viruses. The 5’ nuclease activity of Taq DNA polymerase is used in the real-time reverse transcription PCR assay. The Taq man fast viral 1-step enzyme is a 4× Master mix and five-target primer probe mix that detects influenza A, influenza B, SARS-CoV-2 ORF1ab, respiratory syncytial viruses A/B and actin. When compared with TaqMan ^TM^ and Invitrogen superscript ^TM^ III Platinum and the Meril Kit for SARS-CoV-2, the assay demonstrated 100% sensitivity, specificity, and amplification efficiency of 90.1% for target genes. In conclusion, our one-tube multiplex RT-PCR assay offers a rapid and reliable method for the simultaneous detection of influenza A/B, RSV, and SARS-CoV-2 from nasopharyngeal swabs. This assay has the potential to enhance diagnostic capabilities and improve public health responses during respiratory outbreaks, enabling timely interventions and informed decision making.

## 1. Introduction

The current global health crisis is caused by the severe acute respiratory syndrome coronavirus 2 (SARS-CoV-2) [1]. Diagnostic laboratory testing for respiratory viruses (influenza A/B and RSV) increases during the winter [2]. During flu season, the most common pathogens that cause respiratory infections are respiratory syncytial virus (RSV) and influenza A and B. RSV is more likely to infect children and the elderly [3,4,5]. Influenza A (H1N1) pdm09 and influenza A (H3N2) cause annual epidemics globally.

The influenza viruses A and B cause significant morbidity and mortality worldwide, ranging from simple respiratory infections to lethal pneumonia. Both viruses belong to the Orthomyxoviridae family and share similarities in their structure and mode of transmission. However, there are important differences between influenza A and B viruses in terms of their genetic variability, antigenic characteristics, and impact on public health [6].

Influenza A viruses are known for their high genetic diversity, which is primarily attributed to their ability to undergo frequent antigenic changes through two main mechanisms: antigenic drift and antigenic shift [7]. Antigenic drift refers to gradual changes in the viral surface proteins, hemagglutinin (HA) and neuraminidase (NA), resulting in the emergence of new viral strains that can partially evade pre-existing immunity. Antigenic shift, on the other hand, occurs when two or more different influenza A viruses infect the same host and exchange genetic material, leading to the emergence of novel subtypes with pandemic potential. Influenza A viruses are further classified into subtypes based on the antigenic properties of their HA and NA proteins, with subtypes such as H1N1 and H3N2 being responsible for seasonal influenza outbreaks in humans [8]. In contrast, influenza B viruses undergo antigenic drift at a slower rate compared with influenza A viruses and do not undergo antigenic shift [9]. This means that the genetic changes in influenza B viruses are more gradual and typically result in the emergence of new lineages or strains within the same viral subtype. Although influenza B viruses generally cause milder illness compared with influenza A viruses, they can still cause significant morbidity and mortality, especially in high-risk populations such as young children and the elderly. Detection and differentiation of influenza A and B viruses are crucial for effective surveillance, vaccine development, and clinical management of influenza cases [10]. Various diagnostic methods, including molecular techniques, such as RT-PCR and antigen-based assays, are used to identify and subtype influenza viruses in clinical samples. Understanding the genetic and antigenic characteristics of influenza A and B viruses is essential for monitoring their evolution, predicting their impact on public health, and implementing appropriate preventive and control measures.

While RSV is well known for its role in pediatric respiratory infections, the impact of these viruses on adult respiratory illnesses has not been adequately studied. RSV, on the other hand, is a major cause of morbidity and excess mortality in the elderly and immunocompromised patients [11]. Individuals infected with different respiratory pathogens may exhibit remarkably similar clinical characteristics, making etiological diagnosis difficult. RSV belongs to the Paramyxoviridae family, specifically the genus Orthopneumovirus [12]. RSV is highly contagious and primarily spreads through respiratory droplets when an infected person coughs or sneezes. The virus can survive on surfaces and objects for several hours, facilitating indirect transmission. RSV infections typically occur during the winter months in temperate climates, causing seasonal outbreaks [13]. However, in tropical climates, RSV infections can occur throughout the year [14]. Clinical manifestations of RSV infection can range from mild upper respiratory tract symptoms, such as cough, rhinorrhea, and sore throat, to more severe lower respiratory tract symptoms, including wheezing, respiratory distress, and pneumonia. Infants and young children, particularly those with pre-existing medical conditions, are at higher risk of developing severe RSV disease, necessitating hospitalization and intensive care [15].

Quick diagnostic techniques should enable detailed diagnosis at an early stage of the illness, assisting the treating physician in deciding on the best course of treatment, preventing nosocomial infections, and detecting the start of epidemics [16]. Although patients infected with SARS-CoV-2, influenza A and B, and RSV have similar clinical symptoms, these viruses are treated and managed differently. Unlike common cold viruses, these four infections are frequently accompanied by fever and other systemic symptoms, which can have serious consequences, particularly in the elderly [16].

The WHO GISRS (Global Influenza Surveillance and Response System) encourages the dissemination of information about the currently circulating epidemic viruses. This allows for the improvement in vaccine formulations as well as the early detection of newly circulating influenza, a virus with the potential to spread globally.

To halt the spread of SARS-CoV-2, influenza, and RSV, prompt diagnosis and infection control measures are required. Traditional diagnosis techniques may result in significant delays in the release of final results. The gold standard for the detection of numerous viruses is real-time reverse transcription polymerase chain reaction (rRT-PCR), the most commonly used molecular technique for qualitative diagnostic testing. For many RNA viruses, multiplex reverse transcription polymerase chain reaction (RT-PCR) assay-based diagnosis has been shown to be more rapid and sensitive than traditional viral culture or antigen detection techniques [8]. This test also allows for the co-amplification of multiple target RNA sequences present in a small sample size of the specimen. Real-time PCR improves clinical specimen throughput while lowering screening analytical costs. Multiplex detection of RSV, influenza, and SARS-CoV-2 is critical and widely used [17,18].

In this study, we evaluate the clinical performance of five-target multiplex real-time reverse transcription PCR assays for the simultaneous detection of influenza viruses (A/B), respiratory syncytial viruses (RSV), and SARS-CoV-2.

## 2. Materials and Methods

### 2.1. Design, Setting, and Participants

This was a prospective study conducted between 10 December 2022 and 27 January 2023. The research was carried out at the only Chest Disease Hospital in Srinagar, Jammu and Kashmir, India. We examined 103 clinical swab samples taken from the nasopharyngeal secretions of patients who visited the emergency department (ED) with symptoms of influenza-like illnesses. These samples were collected in 3 mL of Meril Viral transport medium (VTM) and stored at 8 °C. The VTM contains protective proteins, antibiotics, a buffer, and cryoprotectants enabling prolonged storage of samples. The respiratory pathogens and their target genes identified via 5-target assay and TaqMan single target assay kit are described in Table 1.

### 2.2. Ethical Declaration

The study protocol was approved by the institutional review board under IRBGMC/CD32. At the time of the sample collection, informed consent was obtained.

### 2.3. RNA Isolation

After vortexing the sample for 15 s, 200 μL was transferred to a 1.5 mL microcentrifuge tube (MCT). Then, 400 μL of lysis buffer and 10 μL of carrier RNA were added to the MCT and thoroughly mixed. Isopropyl alcohol in the amount of 400 μL was added to the samples, and 500 μL of the total solution was transferred to the spin column in a 2 mL collection tube; the cap was closed and a 1 min centrifuge was performed in a legend micro 21 R at 8000 rpm (Thermo Fisher). After carefully opening the spin column, 500 μL of wash buffer was added and centrifuged at 8000 rpm for 1 min before discarding the filtrate. This procedure was repeated with 500 μL of wash buffer and a 1 min dry spin. Finally, the spin column was placed in a 1.5 mL clear MCT with 60 μL of elution buffer and centrifuged at 8000 rpm for 1 min. For RT-PCR, the eluted viral RNA was stored at −80 °C.

### 2.4. Reverse Transcription Quantitative Real-Time PCR (RT-qPCR)

A 5-target reverse transcription quantitative real-time PCR (RT-qPCR) assay was run on an Applied BiosystemsTM 7500 Fast Dx Real-Time PCR instrument (Thermo Fisher Scientific, Waltham, MA, USA). The 5-target TaqMan assay primer probe mix was used for the qualitative detection of influenza A, influenza B, RSV A/B, ORF 1ab, and a actin. The Taq man fast master mix 4× enzyme (5.0 μL), a primer-probe mix of influenza A/B, RSV A/B, and SARSV-CoV-2 (1.5 μL), RNA sample (7 μL), and RT-PCR grade water were used in the 20 μL RT-PCR reaction (6.5 μL). Finally, to the control addition area, 3.5 μL of a pooled influenza and RSV positive control and 3.5 μL SARS-CoV-2 were added. Negative template control (NTC) and, in addition to positive control extraction (PCE), negative extraction control (NEC) (internal controls) were added to each run.

The primers and probes for influenza A are forward primer 5′- CATGGAATGGCTAAAGACAAGACC-3′, reverse primer 5′- AAGTGCACCAGCAGAATAACTGAG-3′, and the probe 5′-CTGCAGCGTAGACGTTTGTCCAAAATG-3′.

The primer probes for influenza B are BHA-188 forward primer 5′-AGACCAGAGGAAACTATGCCC-3′, reverse primer BHA-347 5′-CTGTCGTGCATTATAGGAAGCAC-3′, and the probe BHA-273 5′-ACCTTCGGCAAAAGCTTCAATACTCCA-3′.

The primer probes for RSV are HRSV forward primer 5′-ATGGCTCTTAGCAAAGTCAAGT3-, reverse primer 5-TGCACATCATAATTRGGAGTRTCA3-, and the probe HRSV 5′- ACACTCAACAAA G’T” CAACTTCTRTCATCCAGCA3- (T residue with black hole quencher BHQ.

The primer probes for ORF1b forward primer are 5′-TGGGGYTTTACRGGTAACCT-3′, reverse primer 5′-AACRCGCTTAACAAAGCACTC-3′, and the probe 5′-CY5-TAGTTGTGATGCWATCATGACTAG -3′.

The thermal cycle was set up for 40 cycles as follows: reverse transcription at 50 °C for 5 min, polymerase activation at 95 °C for 20 s, amplification at 95 °C for 5 s, and data acquisition at 55 °C for 30 s. Cycle threshold (Ct) values cut off ≤ 36 were considered positive.

For reproducibility, the TaqMan assay single-step RT-PCR assay was conducted on BioRad (California, CA, USA) for influenza A and influenza B and the RSV Invitrogen superscript III platinum one-step qRT-PCR assay kit (Thermo Fisher Scientific, Waltham, MA, USA) was used. For SARS-CoV-2, the Meril Assay Kit (orf1b, Ngene, and RnaseP) (Meril, Bruges, Belgium) was used to compare the results obtained from the 5-target assay. For each individual cycle, two positive extraction controls and one negative extraction control was used in addition to a positive control and a negative control in each run.

The cycle conditions are as follows: reverse transcription at 50 °C for 30 min (1 cycle), RT inactivation/denaturation at 95 °C for 10 min (1 cycle), amplification at 95 °C for 15 s followed by 30 s at 55 °C (40 cycles), and data collection (final extension) at 55 °C for 10 min (1 cycle). Furthermore, the RT-PCR cycle conditions for SARS-CoV-2 are as follows: reverse transcription at 50 °C for 15 min; cDNA initial denaturation at 95 °C for 3 min; amplification at 95 °C for 15 s; and data acquisition at 55 °C for 40 s. The assay kits and their fluorophores are shown in Table 1. Ct values ≤35 were considered positive.

## 3. Results

Of the 103 samples, 27 (26.2%) tested positive for influenza A, 5 (4.8%) for influenza B, 18 (17.4%) for RSV, 4 (3.8%) for SARS-CoV-2, and 49 were negative, respectively (Table 2). The four kits showed no significant difference in sensitivity, specificity, or reproducibility. None of the four kits were cross-reactive.

**Figure 1:**
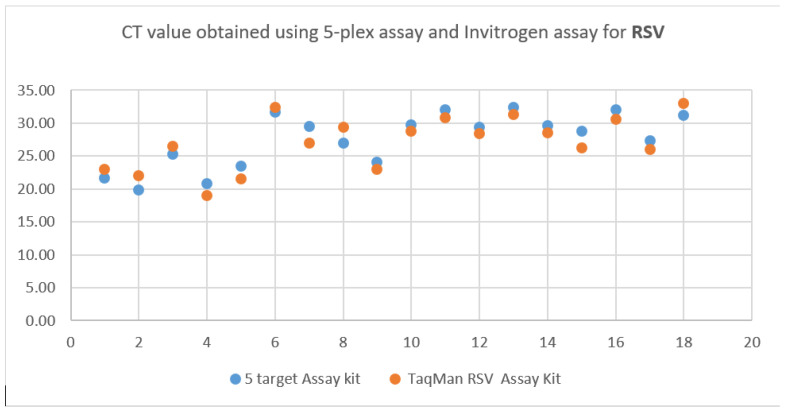
The CT value of RSV (M gene) was obtained during the clinical performance.

**Figure 2:**
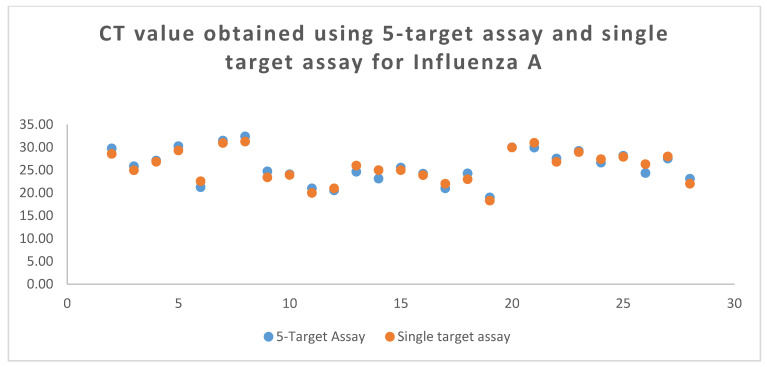
The CT value of influenza A (M gene) was obtained with using 5-plex RT-PCR vs. Single target Assay during the Clinical performance.

**Figure 3:**
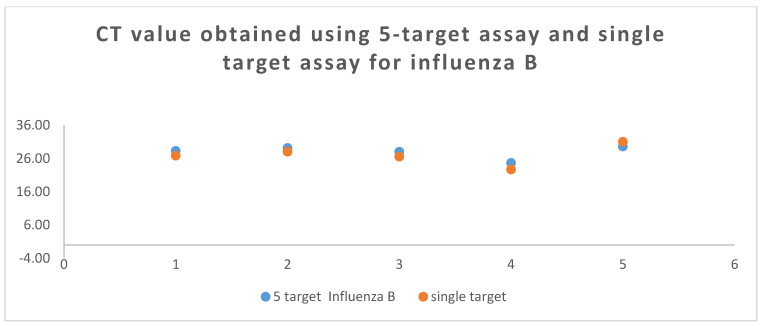
The CT value of influenza B (HA gene) was obtained with using 5-plex RT-PCR vs. Single target Assay during the Clinical performance.

The mean Ct value (positive samples) obtained for influenza A, influenza B, RSV, SARS-CoV-2, and actin was 25.81, 27.92, 27.55, 23.20, and 25.81, respectively. The comparison of Ct values obtained by using different assays is shown in Figure 1, Figure 2 and Figure 3.

**Figure 4:**
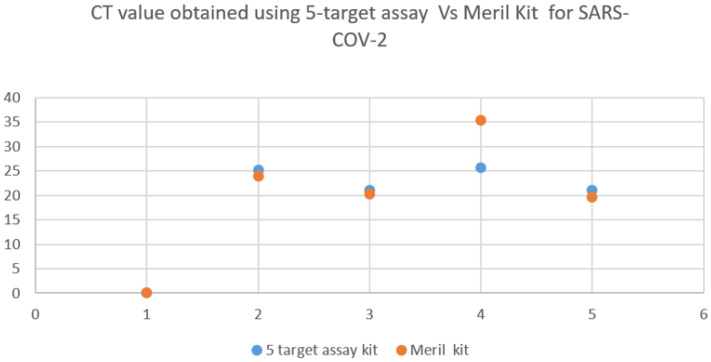
The CT value of SARS-CoV-2 (Orf/N gene) was obtained during the clinical performance.

The sample H07 was detected positive (25.66 Ct) in the five-target assay and the sample was found late positive in the Meril kit assay (35.45 Ct) in Figure 4. No false positives were observed in any of the four assay kits. There were no cases of influenza A, B, or RSV in the SARS-CoV-2 positive samples and no SARS-CoV-2 positive cases in the samples that tested positive for influenza A/B or RSV. Influenza A showed the highest positivity followed by RSV, influenza B, and SARS-CoV-2, respectively. We collected samples from symptomatic patients throughout the same influenza season in order to account for false-positive outcomes. The positive and negative results from the five-target assay were reproducible while being tested on the other three assay kits. Second, the addition of positive and negative extraction controls (PEC and NEC) in each run confirmed the samples as true positives and negatives. The Supplementary Material at https://zenodo.org/record/7903681 (accessed on 25 May 2021).

## 4. Discussion

Traditional diagnostic methods for respiratory viral infections often rely on individual singleplex assays, necessitating separate reactions for each virus of interest [19]. This approach is time-consuming, labor-intensive, and requires a significant amount of clinical sample material. Moreover, during the ongoing COVID-19 pandemic, the simultaneous detection of multiple respiratory viruses has become even more critical, as co-infections or differential diagnoses between these viruses can have profound implications for patient care and public health interventions. To address these challenges, the development of multiplex assays capable of simultaneously detecting multiple respiratory viruses in a single reaction has gained considerable attention. The integration of RT-PCR with multiplexing strategies offers a promising solution by enabling the amplification and detection of multiple viral targets concurrently [20].

The primary objective of this study was to assess the performance and reliability of our multiplex RT-PCR assay using a panel of known positive and negative clinical samples. We compared the results obtained from the multiplex assay with those obtained from individual singleplex RT-PCR assays to validate its accuracy and sensitivity. Additionally, we evaluated the assay’s performance in a real-world setting by testing nasopharyngeal swabs from patients presenting with respiratory symptoms, where a timely and accurate diagnosis is crucial for appropriate patient management and infection control.

Our findings show that a five-target assay kit test is a sensitive method for detecting all five respiratory viruses (influenza A/B, RSV, SARS-CoV-2, and actin. We were able to detect all five viral RNA targets in a single tube reaction, which is more efficient, has a higher throughput, and has a turnaround time of about 3.5 h when compared with performing individual RT-PCR for each respiratory virus using the other three kits. The five-target RT-PCR method detects RSV, SARS-CoV-2, influenza viruses (A/B), and actin with a high degree of accuracy. The ABI 7500 Fast Dx Real-Time PCR instrument is capable of detecting numerous dyes (FAM, VIC, JUN, and NED) with diverse emission wavelengths; the specific primer-probe mix, for example, can be tagged with various fluorogenic dyes to discriminate between influenza viruses A and B, RSV, and SARS-CoV-2 [21]. Inadequate sampling results in a false negative result. In the five-target assay, the human housekeeping gene measures the quality of the sampling and hence detects the pre-analytical error. The subtype influenza virus A/B and RSV A/B can be performed in-depth, utilizing subtype-specific primers. Subtyping has been performed using RT-PCR in previous studies. During the flu season (winter), it is critical to identify viral respiratory infections quickly and accurately. Clinical samples with a shorter turnaround time result in better patient care. In previous studies, RT-PCR was used to subtype the samples [22,23,24,25]. The rapid multiplex five-target assay also has the advantage of a standardized protocol, and amplification can be performed under uniform conditions. The likelihood of contamination is also reduced in multiplex assays compared with individual RT-qPCR identifications of respiratory viruses. Taq man RT-qPCR is more sensitive than traditional PCR at detecting a small number of RNA copies [21].

The respiratory viruses that cause the flu (influenza A/B) are particularly risky in the elderly and infants/children. The severity of the disease may necessitate hospitalization. Sensitive and rapid molecular diagnostics for respiratory viral pathogens are critical for preventing nosocomial transmission and limiting antibiotic use [26]. The multiplex assay detected SARS-CoV-2, influenza A, and influenza B with sensitivity levels of 98.1%, 97.67%, and 100%, respectively [27]. The most common co-infections were rhinovirus/enterovirus, RSV, and non-SARS-CoV-2 Coronaviridae. Previous research found that while 26.7% of samples tested negative for SARS-CoV-2, 20.7% of those who tested positive for SARS-CoV-2 also tested positive for one or more other infections [28]. Since infection prevention and control measures must be put into place quickly and hospital departments have been overburdened during the pandemic’s multiple waves, the time to result is difficult for clinical laboratories, especially for COVID-19 diagnosis.

Aerosols emitted by infected people can spread the disease when people come into close, unprotected contact with them [29,30]. To aid in the management of viral respiratory infections, technologies for rapid and accurate infection detection and analysis are constantly being developed. To effectively manage and control the disease in the general population, the SARS-CoV-2 infection must be identified quickly, completely, and accurately [31,32,33]. An RT-sensitivity PCR is dependent on pre-analytical factors such as sampling, particularly for samples with a Ct value greater than 35.0; furthermore, poor sampling is associated with poorer results [32]. The five-target assay of SARS-CoV-2/Flu/RSV amplified actin, allowing us to assess the quality of the sampling. Kuroiwa et al. [33] performed the immunochromatographic assay (ICA) detection test for respiratory pathogens and when compared to RT-PCR it was found to be more sensitive in detecting respiratory viruses in clinical samples [34].

Immunochromatographic assays (ICAs), also known as lateral flow assays or rapid diagnostic tests, utilize antigen–antibody interactions to detect specific viral proteins in clinical samples. These tests offer several advantages, including rapid results (within minutes), simplicity of use, portability, and minimal technical requirements. ICAs are well suited for point-of-care testing, decentralized settings, resource-limited areas, and rapid screening during outbreaks. However, their sensitivity and specificity may vary depending on the specific assay design and target antigen. RT-PCR, on the other hand, has been the gold standard for diagnosing respiratory viral infections. It detects the viral genetic material by amplifying and detecting specific RNA or DNA sequences. RT-PCR offers high sensitivity and specificity and is capable of detecting low viral loads and differentiating between viral strains. However, it requires specialized laboratory equipment, skilled personnel, and longer turnaround times, making it less suitable for rapid point-of-care testing.

The primary strategy of this assay is to provide an accurate result in order to avoid a false negative result caused by insufficient sampling. The five-target RT-qPCR is a necessary post-COVID-19 addition to target all respiratory viruses at once. This multiplex is accurate and precise, and detection of coinfection is the advantage.

SARS-CoV-2 was not tested for cross-reactivity with any other coronaviruses; therefore, this study has few limitations. Secondarily, while the five-target kit was used as a comparison kit in this study, it did not quantify the co-infection rates of SARS-CoV-2 and other respiratory viruses and subtyping of influenza A and lineages of influenza B.

## 5. Future Perspectives

The simultaneous detection of influenza A/B, respiratory syncytial virus (RSV), and SARS-CoV-2 in nasopharyngeal swabs via a one-tube multiplex reverse transcription polymerase chain reaction (RT-PCR) assay holds great promise for improving respiratory virus diagnostics. The development of user-friendly, portable, and rapid detection platforms, such as paper-based assays or handheld devices, could enable decentralized testing, especially in resource-limited or remote settings.

Future research could focus on expanding the panel to include other clinically relevant respiratory pathogens. Incorporating additional targets, such as other influenza strains and common respiratory viruses (e.g., rhinovirus and adenovirus), would provide a more comprehensive diagnostic tool for respiratory infections. Incorporating host gene expression profiles or immune response markers could potentially enhance diagnostic accuracy and aid in distinguishing between viral and non-viral respiratory infections, as well as assessing disease severity and monitoring treatment response.

## 6. Conclusions

The five-target kit is a quick, highly sensitive, and specific quantitative real-time PCR to detect influenza viruses A/B, SARS-CoV-2, RSV, and actin at the same time. Results can be received in 3.5 h, providing enough time for proper clinical management, turnaround time, and antiviral therapy evaluation.

## Figures and Tables

**Table 1:** RT-PCR of the evaluated assays and Fluorophores.

Kit Method	Method	Sample Volume	Viral Genes Target and Fluorophores			
			SARS-CoV-2	RSV	Influenza A	Influenza B
5-target assay Taqman	RT-PCR	400 μL	Orf1b (Cy5)	M * (FAM)	M (VIC)	HA ** (NED)
Meril kit	RT-PCR	200 μL	ORF1b/N (FAM/HEX)	-	-	-
TaqMan assay kit Influenza A	RT-PCR	200 μL	-	-	M (FAM)	-
TaqMan assay kit Influenza B	RT-PCR	200 μL	-	-	-	HA (FAM)
Invitrogen assay kit (RSV)	RT-PCR	200 μL	-	M (VIC)	-	-

** Hemagglutinin (HA); * Matrix (M); orf1b/N open reading frame and nucleocapsid.

**Table 2:** Results for influenza A, influenza B, RSV, and SARS-CoV-2 viruses (*n* = 103).

Virus	Target Gene	Five-Assay Target Assay Kit	TaqMan Assay Kit	TaqMan Assay Kit	Invitro Assay Kit	Meril Kit
Influenza virus A	M	27/103	27/103	-	-	-
Influenza virus B	HA	5/103	-	5/103	-	-
RSV	M	18/103	-	-	18/103	-
SARS- CoV-2	ORF 1ab/ N *	4/103	-	-	-	4/103

* The orf1b/N gene target was detected in the Meril Kit and only orf1b was detected in the five-target assay.

## Data Availability

The data presented in this study are available on request from the corresponding author.

## Supplementary Materials

The following supporting information can be downloaded at: https://zenodo.org/record/7903681.
