# Supplementary material for: Simultaneous Detection of Influenza A/B, Respiratory Syncytial Virus, and SARS-CoV-2 in Nasopharyngeal Swabs by One-Tube Multiplex Reverse Transcription Polymerase Chain Reaction
Source: Trop Med Infect Dis. 2023 Jun 19;8(6):326. doi: 10.3390/tropicalmed8060326 (PMC10305207; doi:10.3390/tropicalmed8060326)
Supplement: Supplementary file 1 [file tropicalmed-08-00326-s001.zip › tropicalmed-2385844-supplementary.pdf]

Table S1. Confirmed Influenza A positive cases and their CT values were obtained by using different kits.

			5-target assay		TaqMan Assay kit	
Sample ID	Age	Gender	Ct Value	Result	Ct Value	Result
A03	70	M	29.76	Positive	28.56	Positive
A04	60	M	25.84	Positive	24.96	Positive
A05	40	M	27.10	Positive	26.84	Positive
A06	65	M	30.26	Positive	29.31	Positive
A07	45	F	21.24	Positive	22.56	Positive
A08	65	F	31.47	Positive	30.95	Positive
A09	85	M	32.39	Positive	31.25	Positive
A10	18	M	24.72	Positive	23.41	Positive
A11	60	F	24.09	Positive	23.94	Positive
A12	60	F	21.00	Positive	20.00	Positive
B01	50	F	20.57	Positive	21.00	Positive
B02	52	F	24.65	Positive	26.00	Positive
B03	56	F	23.12	Positive	25.00	Positive
B04	40	M	25.57	Positive	24.98	Positive
B05	35	F	24.23	Positive	23.89	Positive
B06	69	F	21.00	Positive	22.00	Positive
B07	56	M	24.30	Positive	23.00	Positive
B08	23	F	19.00	Positive	18.30	Positive
B09	71	F	30.01	Positive	29.96	Positive
B10	65	F	29.94	Positive	31.00	Positive
B11	80	F	27.56	Positive	26.80	Positive
B12	65	F	29.21	Positive	28.95	Positive
C01	23	F	26.64	Positive	27.40	Positive
C02	59	M	28.14	Positive	27.90	Positive
C03	45	F	24.35	Positive	26.30	Positive
C04	70	M	27.53	Positive	28.00	Positive
C05	45	F	23.10	Positive	22.00	Positive

Table S2. Confirmed Influenza B-positive cases and their CT values were obtained by using different kits.

			5 target Assay kit		TaqMan Assay kit	
Sample ID	Age	Gender	Ct value		Ct value	Result
D01	63	M	28.24	Positive	26.84	Positive
D04	43	M	29.13	Positive	28.02	Positive
D10	59	F	28.00	Positive	26.53	Positive
D11	49	M	24.64	Positive	22.67	Positive
D12	74	M	29.56	Positive	31.02	Positive

Table S3. Confirmed SARS-COV-2 positive cases and their CT values were obtained by using different kits.

			5 target assay kit		Meril kit	
Sample ID	Age	Gender	Ct Value	Result	Ct value	Result
H05	57	F	25.15	Positive	24	positive
H06	66	F	21.00	Positive	20.32	positive
H07	41	M	25.66	Positive	35.45	*late positive
H08	35	F	21.00	Positive	19.56	positive

Table S4. Confirmed RSV-positive cases and their CT values were obtained by using different kits.

Sample ID	Age	Gender	5 target Assay kit Ct value	Result	SuperscriptIII platinum Assay Kit Ct value	Result
E12	66	M	21.62	Positive	23.02	Positive
F01	49	M	19.90	Positive	22	Positive
F02	41	M	25.27	Positive	26.52	Positive
F03	58	M	20.77	Positive	19.05	Positive
F04	56	F	23.52	Positive	21.56	Positive
F05	39	F	31.63	Positive	32.4	Positive
F06	69	M	29.49	Positive	27	Positive
F07	58	M	27.03	Positive	29.35	Positive
F08	72	M	24.12	Positive	23	Positive
F09	58	M	29.77	Positive	28.75	Positive
F10	36	F	32.00	Positive	30.87	Positive
F11	71	F	29.36	Positive	28.4	Positive
F12	45	F	32.36	Positive	31.3	Positive
G01	69	F	29.63	Positive	28.49	Positive
G02	56	M	28.84	Positive	26.21	Positive
G03	57	F	32.00	Positive	30.56	Positive
G04	63	M	27.35	Positive	26	Positive
G05	75	M	31.20	Positive	33.02	Positive

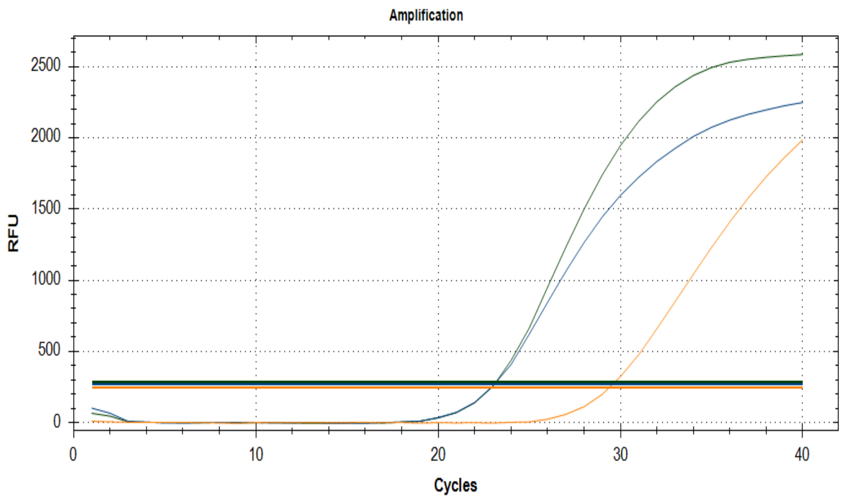

Figure S1. Bio-Rad CFX96 detecting the dyes (FAM,HEX and ROX) for SARS COV-2 (Meril kit)

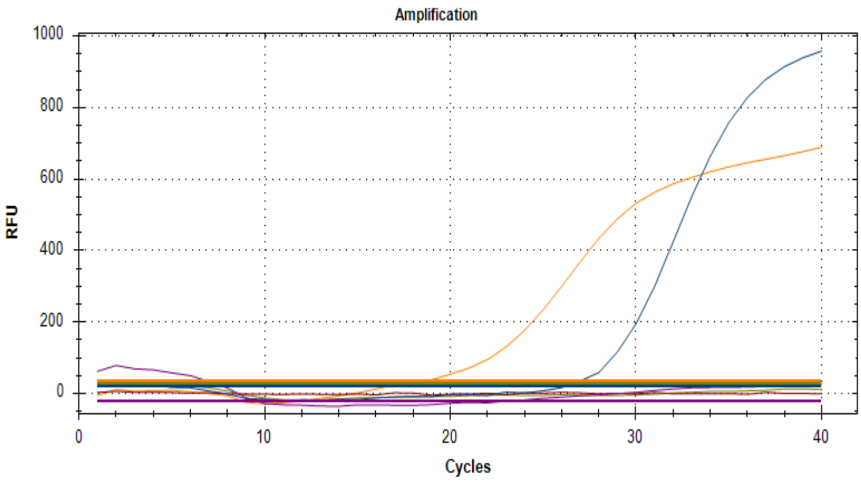

Figure S2. Bio-Rad CFX96 detecting the dye FAM for RSV (TaqMan target Assay Kit)

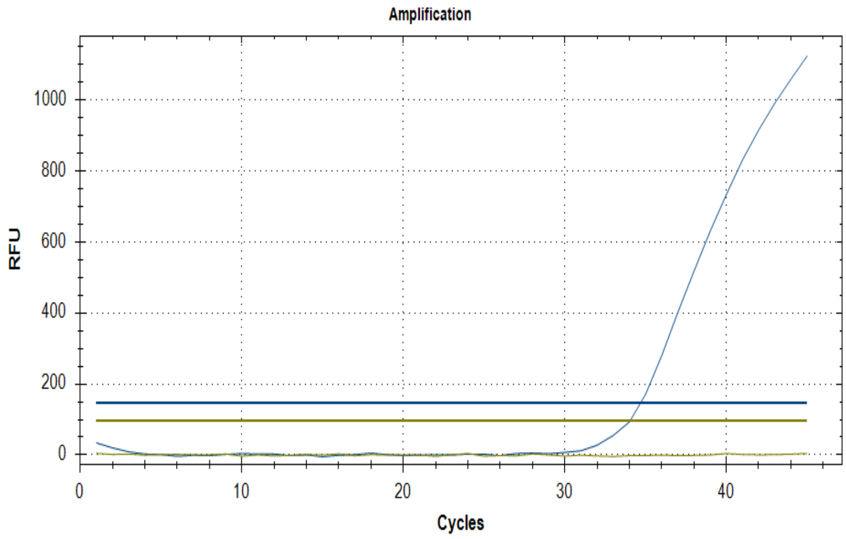

Figure S3 Bio-Rad CFX96 detecting the dye FAM for Influenza A (TaqMan target Assay Kit)

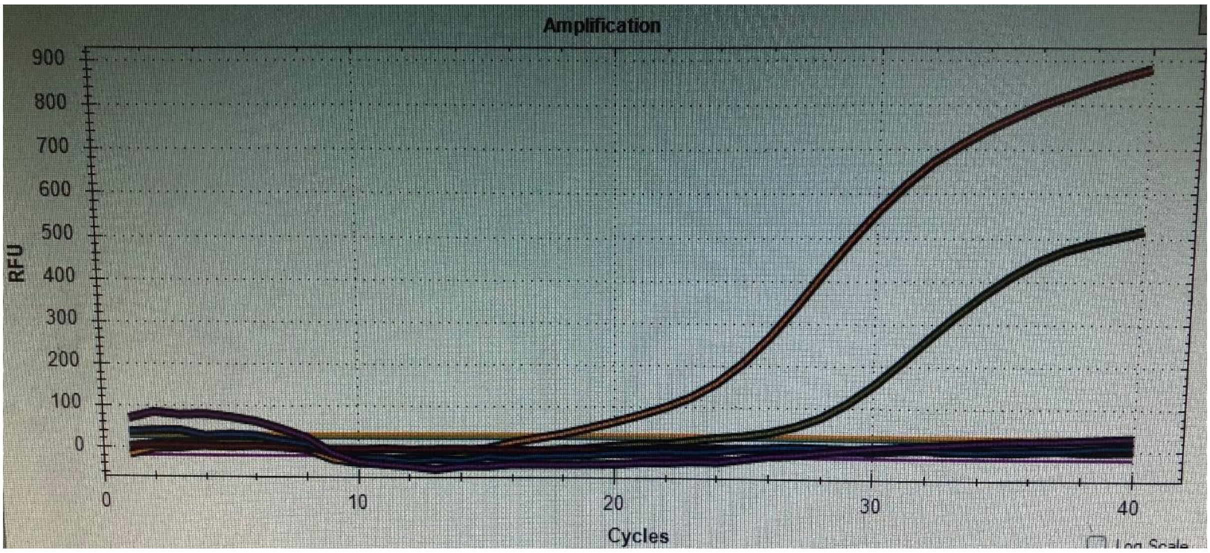

Figure S4 Bio-Rad CFX96 detecting the dye VIC for Influenza B and ROX dye detecting RNaseP (TaqMan target Assay Kit)

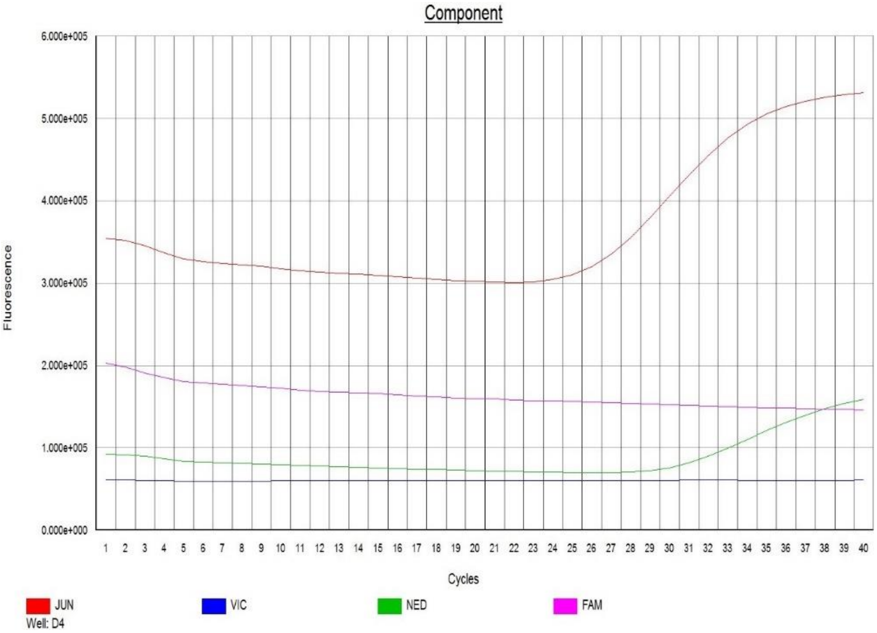

Figure S5 ABI 7500 Fast Dx Real-Time is capable of detecting dye (NED) for Influenza B (multiplex target kit)

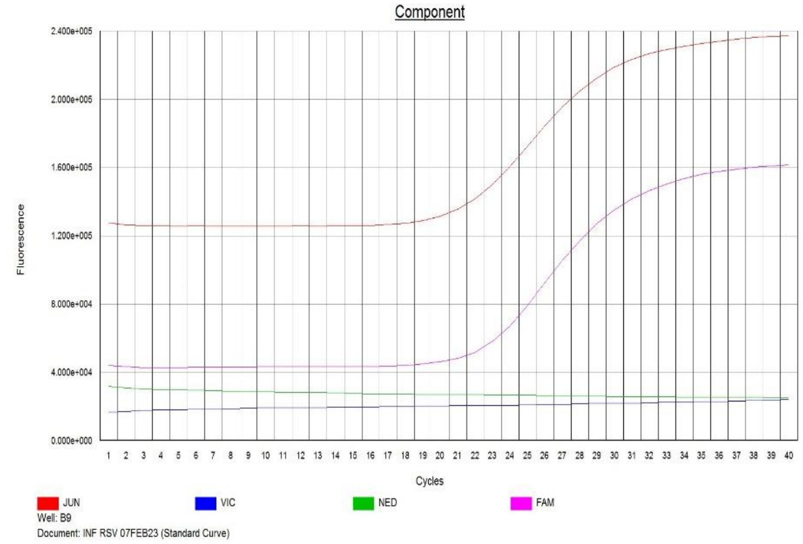

Figure S6 ABI 7500 Fast Dx Real-Time is capable of detecting dye (FAM) for RSV (multiplex target kit)

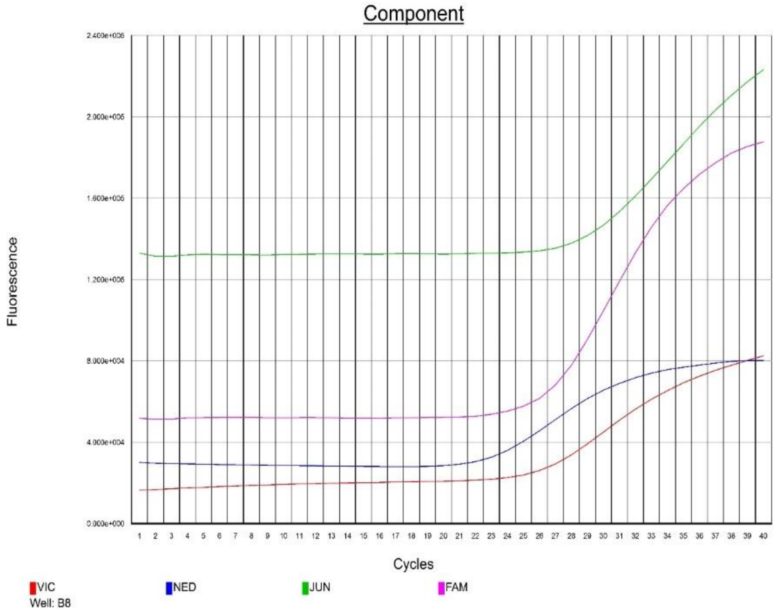

Figure S7 ABI 7500 Fast Dx Real-Time detecting numerous dyes (FAM, VIC, JUN, NED) (multiplex target kit) JUN ; Actin, FAM; RSV, NED; InfB, VIC; Inf A
